# Selenium: An Antioxidant with a Critical Role in Anti-Aging

**DOI:** 10.3390/molecules27196613

**Published:** 2022-10-05

**Authors:** Geir Bjørklund, Mariia Shanaida, Roman Lysiuk, Halyna Antonyak, Ivan Klishch, Volodymyr Shanaida, Massimiliano Peana

**Affiliations:** 1 Council for Nutritional and Environmental Medicine (CONEM), Toften 24, 8610 Mo i Rana, Norway; 2 I. Horbachevsky Ternopil National Medical University, 46001 Ternopil, Ukraine; 3 Department of Pharmacognosy and Botany, Danylo Halytsky Lviv National Medical University, 79010 Lviv, Ukraine; 4 CONEM Ukraine Life Science Research Group, Danylo Halytsky Lviv National Medical University, 79010 Lviv, Ukraine; 5 Department of Ecology, Ivan Franko National University of Lviv, 79005 Lviv, Ukraine; 6 Design of Machine Tools, Instruments and Machines Department, Ternopil Ivan Puluj National Technical University, 46001 Ternopil, Ukraine; 7 Department of Chemical, Physics, Mathematics and Natural Sciences, University of Sassari, 07100 Sassari, Italy

**Keywords:** selenium, health benefit, human aging, antioxidant effect, immunoprotection, chemoprevention

## Abstract

Aging is characterized by an imbalance between damage inflicted by reactive oxygen species (ROS) and the antioxidative defenses of the organism. As a significant nutritional factor, the trace element selenium (Se) may remodel gradual and spontaneous physiological changes caused by oxidative stress, potentially leading to disease prevention and healthy aging. Se is involved in improving antioxidant defense, immune functions, and metabolic homeostasis. An inadequate Se status may reduce human life expectancy by accelerating the aging process or increasing vulnerability to various disorders, including immunity dysfunction, and cancer risk. This review highlights the available studies on the effective role of Se in aging mechanisms and shows the potential clinical implications related to its consumption. The main sources of organic Se and the advantages of its nanoformulations were also discussed.

## 1. Introduction

Aging is an important risk factor in the advancement of many age-related disorders [1]. Since there is a steady trend toward the aging of the human population on a global scale, the number of diseases associated with aging is also gradually increasing. The pathogenesis of various health disorders, including neurodegenerative diseases or cancer, is due to the accumulation of reactive oxygen species (ROS) that cause oxidative stress and inflammation, which are the main contributors to cellular senescence [1,2]. Generally, aging is characterized by an imbalance between damage inflicted by ROS and the organism’s antioxidant defenses [3]. Free radicals could be produced due to the influence of environmental pollutants, metal ions, radiation, or by-products of metabolized drugs [4]. ROS production and oxidative damage to biomacromolecules (nucleic acids, lipids, and proteins) can represent a suitable environment for developing age-related diseases [5,6]. Endogenous defense mechanisms of the human body cannot supply the full prevention of ROS damage, and different natural sources of dietary antioxidants are especially important for this purpose [7]. The antioxidant properties of many polyphenols, vitamins, and trace elements are well known [4,7,8,9,10,11]. The deficiency of micronutrients such as vitamins and minerals can suppress immunity and cause a predisposition to infectious diseases, cancer, neurodegeneration, cardiovascular disorders, and hormonal misbalance [12]. Natural antioxidants, as effective free radical scavengers, are indispensable in preventing and treating many age-related disorders [1,4].

The trace element selenium (Se) was regarded as a dietary supplement for improving health as it possesses valuable antioxidant properties [13]. It may remodel gradual and spontaneous biochemical and physiological changes, potentially leading to disease prevention and healthy aging, because it is involved in improving antioxidant defense, immune functions, and metabolic homeostasis [14]. Se is naturally found in water, soil, and food [15]. Amino acids, peptides, and enzymes are regarded as the main biologically important Se-containing organic compounds [13]. Selenocysteine, the S-to-Se substituted variant of amino acid cysteine, is a key component of selenoproteins [16]. Thus, the physiological effect of Se is mainly due to its incorporation in selenoproteins [17].

It should be noted that 25 selenoproteins have been discovered in humans [18]. Selenoproteins have been implicated in many metabolic and functional pathways, such as aging, cancer, or infection [19]. Thus, the Se-containing enzyme glutathione peroxidase can help lower free radical reactions to tolerable levels by reducing H_2_O_2_ to H_2_O and organic hydroperoxides (ROOH) to alcohol (ROH) [20,21]. The Se-dependent glutathione peroxidases (GPX1–4 and GPX6) and thioredoxin reductases (TrxR1-3) directly suppress oxidative stress; cytosolic GPX4 is essential for embryonic development and cell survival. GPX1 is the most abundant selenoprotein and a major metabolic form of body Se against severe oxidative stress [22]. Incidentally, GPX1 was the first selenoprotein discovered in the mammalian body [17]. GPX1 is a special mammalian selenoenzyme maintaining redox equilibrium by detoxifying ROS [17]. Several non-enzymatic selenoproteins are also known, such as selenoproteins F, H, I, K, etc. [17]. Avery and Hoffmann reported that Se deficiency could cause immune incompetence, significantly increasing susceptibility to pathogens or even cancer [17]. For instance, the health-promoting functions of selenoprotein K were proven in the example of the immune system functioning [23].

According to the WHO recommendations, the daily intake of Se by adults should be at 40–70 µg/day depending on gender and body condition (weight, pregnancy status in women, etc.) [24]. Nevertheless, the average content of Se in the daily diet quite often does not reach this level. The typical level of daily consumption varies in the range of 30–50 µg/day in different European countries [20]. It should be mentioned that Se in doses above 400 µg/day shows harmful actions. The uncontrolled intake of Se-enriched products may result in poisoning [20]. Therefore, Se could be considered a trace element characterized by a very narrow range of concentrations in the human body between deficiency, optimal physiological, and toxic levels [20]. The benefits of Se for the human body are most often evaluated during studies of its content in biological material (blood, plasma, urine, tissue) or glutathione peroxidase activity [25]. The normal range for plasma Se is about 120–160 ng/mL [26].

**Figure 1:**
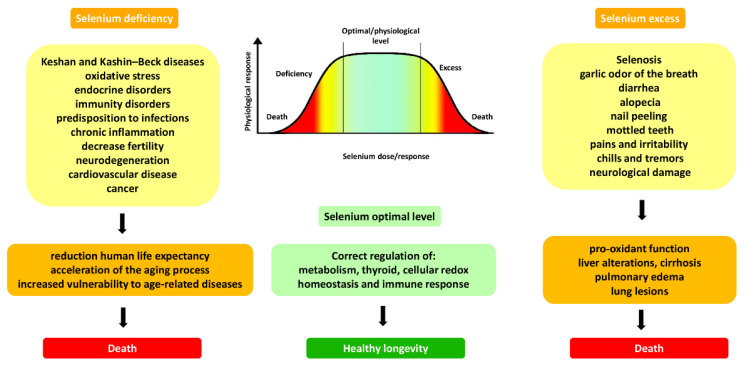
The main outcomes of Se deficiency and excess.

Se deficiency, which affects about one billion people worldwide, may significantly affect health [27]. In many countries, Se may be deficient because of its low concentration in the soils and, accordingly, in the plants that grow on this substrate [13]. Such famous endemic Asiatic diseases as Keshan and Kashin–Beck are due to Se deficiency. It should be mentioned that more than 50% of Chinese regions are characterized by Se deficiency in the soil [28]. Se has a narrow margin between its essential levels and the amounts associated with toxicity [13]. Generally, Se’s health effect (beneficial or toxic) is dose-dependent and related to the chemical form of this trace element and its bioavailability [29]. Slight excess in Se content can result in toxicity; thus, it must be taken carefully and cautiously [20,30]. Health outcomes occur when there is a lack or excess of Se in the body (Figure 1). In particular, Se deficiency leads to endocrine and immunity disorders, infections, chronic inflammation, neurodegeneration, cardiovascular disease, and cancer, which ultimately negatively affect longevity [31].

The excess of Se in some geographic regions can cause another disorder called ‘selenosis’ [13,32]. Clinical signs of selenosis include garlic odor of the breath and diarrhea, alopecia, nail peeling, pains, irritability, chills, tremors, and neurological damage. Extreme selenosis cases can show liver alterations, cirrhosis, pulmonary edema, lung lesions, or death [33].

The main form of Se ingested by humans is regarded as selenomethionine [17]. It is worth noting that organic selenomethionine could cause toxicity at concentrations much higher than inorganic Se [34]. Organic Se, especially SeMet, the major food species, is more beneficial than its inorganic form in the frame of a balanced diet [29].

This element, taken in excess as dietary supplementation or as a result of environmental pollution, may have a toxic effect in the form of joint diseases and disorders of the blood system [35]. Toenail health demonstrates Se’s nutritional status in epidemiological studies more significantly than other biomarkers [31].

The risk of Se deficiency seems to increase in proportion to age [36] and age-related diseases [22]. Se can be considered a longevity indicator in an elderly population since it plays a role in health maintenance in aging individuals [37]. Inadequate Se status may reduce human life expectancy by accelerating the aging process or increasing vulnerability to various age-related diseases. González et al. showed that maintaining good serum Se levels is important since it may affect the self-perception of health, physical activity, and, consequently, the quality of life in older adults [38]. This review highlights the available studies on the effective role of Se in aging mechanisms and shows the potential clinical implications related to its consumption. The main sources of organic Se were also discussed.

## 2. The Role of Se in the Prevention and Treatment of Health Disorders

### 2.1. Oxidative Stress, Inflammation, and Immunity

In the inevitable aging process provided by nature, there is an imbalance between antioxidative defense and ROS, irreversible changes in mitochondrial renewal, and stem cell exhaustion [39]. According to Alehagen et al., these disorders are closely associated with chronic inflammation, which accompanies age-related diseases [39]. Simonoff et al. claimed that the antioxidant status of older people could be evaluated by measuring blood Se and vitamin (A and E) levels [40]. Serum and plasma Se levels, glutathione peroxidase activity, and selenoprotein P concentrations are commonly used measures of a Se status in humans [17].

Se possesses antioxidant, immunostimulating, and anti-inflammatory effects [33]. Many selenoproteins are involved in the regulation of antioxidant activities [41]. The selenocysteine residue in selenoprotein K is regarded to be responsible for its bioactivity [23]. Selenoproteins such as GPXs 1–4 efficiently detoxify cellular peroxides that protect against ROS [13]. In vivo studies have shown that in mice treated with an abdominal injection of D-galactose to induce the aging model, selenoproteins extracted from Se-rich rice enhanced the enzymatic antioxidant capacity (GSH-Px and SOD) in the liver and serum of mice of Se-diet group, compared to the control group [42].

As a cofactor of enzymes involved in antioxidant protection, Se plays a significant role in regulating different inflammatory processes in the organism [35]. Insufficient Se level in the organism is associated with such inflammatory skin diseases as psoriasis and atopic dermatitis [43]. Generally, Se stimulates increasing antibody production in the immune system [20,44]. The optimal Se status (60–175 ng Se/mL plasma) can mitigate an inflammatory process and reduce complications in the lungs, intestines, etc. [17]. Espaladori et al. found that Se used for intracanal medication could potentiate the healing effects of calcium hydroxide in the anti-inflammatory response in periapical dental tissues [45]. According to Ceyan et al., Se demonstrated the protective effect against oxidative toxicity induced by dental amalgam [46].

A group of 16 centenarians, aged 101 to 105 years, living in the Upper Silesia district (Poland), had significantly higher red blood cell glutathione reductase and catalase activities than young, healthy female adults [47]. The increase in the activities of enzymes in the blood of the centenarians, mainly glutathione reductase and catalase, could be interpreted as a favorable response to oxidative stress, which enables the balance between the production of ROS and efficacy of antioxidant defenses in normal aging [47]. Se supplementation in respiratory distress syndrome patients can also modulate the inflammatory response by restoring the antioxidant capacity of the lungs through interleukin (IL)-1β and IL-6 levels [48]. Selenoproteins are very important for prostanoid metabolism due to their immunomodulatory effects [49].

Se deficiency has a negative effect on the condition of innate and adaptive immune responses [17]. The impact of Se on the immune system is multitargeted, i.e., modulating the activities of neutrophils, macrophages, natural killer cells, and T and B-lymphocytes [18]. The studies of the last decade showed that optimal Se supplementation could be used to effectively support the immune system during childhood leukemia [50]. The sufficient Se level improved the phagocytosis functions in macrophages and T-cells activity [51].

Many selenoenzymes and non-enzymatic selenoprotein K, an endoplasmic reticulum transmembrane protein important for calcium-dependent signaling, play an important role in the activation of immune cells [18,52]. Marciel and Hoffmann reported that calcium homeostasis might be altered when different types of cells transform into tumor cells. Selenoprotein K, playing a prominent role in regulating immunity as a cofactor for an enzyme involved in post-translational modifications and maturation of proteins in the endoplasmic reticulum, promotes the calcium flux in the endoplasmic reticulum of immune cells [23]. Thus, Se deficiency is related not only to disrupted antioxidant defense but also to calcium flux and protein folding in cells [53]. It could be concluded that Se supplementations can boost immunity against cancer or other diseases, creating an opportunity for healthy longevity.

### 2.2. Infections

As it is known, ROS are frequently produced in the human body during viral infections, and their excess can induce oxidative stress being one of the hallmarks of clinical symptoms for many diseases [54]. Among the essential micronutrients implicated in the progression of viral infection, Se plays an important role in redox homeostasis and antioxidant defense due to its incorporation, as already mentioned, in the vitally important selenoproteins [55,56]. For example, Se supplementation strongly affected viral suppression and T-cell recovery in HIV-infected patients in Rwanda [57]. Patients infected with tuberculosis and HIV had lower Se statuses when compared to healthy humans [17].

For the third year, the coronavirus disease COVID-2019 has been affecting millions of people and leading to periodic global pandemic outbreaks. A deficiency in Se was recorded in several health disorders caused by viruses, including COVID-19 [35,55]. Recent studies revealed that COVID-19 patients have lower circulating levels of iron (Fe), zinc (Zn), and Se [58]. Se supplementation in COVID-19 patients has been useful in preventing disease progression [59]. Hargreaves and Mantle found that coenzyme Q10 and Se could decrease oxidative stress and inflammation levels in COVID-19-infected patients [60]. Se-containing agents were regarded as important regulators of the defense mechanisms against COVID-19 due to their anti-inflammatory and immunomodulatory properties [61].

Antibiotic resistance and cancer are now considered the two most significant public health problems that are leading to significant mortality of the planet’s population due to the deaths of over 1.5 million people per year [62]. This suggests that antibiotics and available chemotherapeutics do not supply effective treatment for such serious health problems. Se nanoparticles demonstrated significant antibacterial activity against multidrug-resistant bacteria [63]. Se-containing sealant eliminated the biofilm formation in dental plaque caused by pathogenic bacteria *Streptococcus mutans*, *S. sanguinis*, and *S. salivarius* [64,65]. Seguya et al. compared the efficacy of an organoselenium sealant and chlorhexidine diacetate to prevent the formation of *S. mutans* biofilm on human teeth [66]. It was reported that chlorhexidine diacetate and organoselenium slightly inhibited *S. mutants’* attachment to the teeth [66].

Se supplementation was beneficial during murine infection with a Brazil strain of *Trypanosoma cruzi*, resulting in decreased parasitemias and increased longevity. After 64 days of infection, groups receiving 4 ppm and 8 ppm Se as sodium selenate in drinking water exhibited 60% survival, and the group without Se demonstrated 0% survival [67].

### 2.3. Endocrine System Disorders

As it is known, the proper functioning of the thyroid gland requires several elements, including Se, Zn, and copper (Cu), in addition to iodine [68]. Se is one of the important regulators of metabolic processes, and optimal intake is necessary to maintain homeostasis [69].

As reported recently, 35 selenoproteins have been identified [70]. As it was noted by Schomburg [71], a significant number of selenoproteins are involved in the functioning of the thyroid gland. Se deficiency is crucial for developing Hashimoto’s thyroiditis and Graves’ disease [72]. A severe deficiency of Se in diet during pregnancy can cause the development of autoimmune thyroid disease [73].

Three of them are iodothyronine deiodinases, which play a key role in thyroid hormone metabolism. One of the critical roles of Se-enzymes is the involvement in the thyroid hormone synthesis and, therefore, in regulating basic metabolism in all body cells and tissues [74]. Iodothyronine deiodinases cleave iodine-carbon bonds in the thyroid hormone metabolism. Selenoproteins P and GPX 3, present in the human plasma, are often estimated as biomarkers for assessing the Se status of an organism [13,34]. Administration of Se in the case of autoimmune thyroiditis reduced autoimmune antibody titers and improved the well-being of patients [75].

Persistent Se deficiency may also cause infertility [35]. Many clinical studies implicate Se deficiency in several reproductive complications, such as male and female infertility, miscarriage, preterm labor, etc. [76,77].

As was concluded by Prabhu and Lei, both Se deficiency and oversupply seem to dysregulate glucose metabolism and potentiate the risk of type 2 diabetes in several animal studies, with less clear associations discovered in clinical studies [34].

### 2.4. Cancer

During the 5-year epidemiologic studies and clinical trials conducted by Japanese scientists, the significant effects of sufficient Se status were established in patients with different cancer types [78]. Razaghi et al. reported that nutritional doses of Se can stimulate the immune system against cancer [18]. The anti-cancer effects of selenoprotein K have been revealed in melanoma models in vivo and in human melanoma cell lines [23]. According to Varlamova et al., various mechanisms are involved in the anticarcinogenic effect of Se. Besides the prominent antioxidant effects, Se-containing compounds can maintain DNA stability, regulate inflammatory and immune responses, and inhibit the toxicity of heavy metals [79]. Thus, Se possesses anticarcinogenic properties, especially if administered in a preventive manner before the onset of a disease or at the early stage of its development [20,56,80]. However, its overdosing can act as pro-oxidant inducing cell death. As it is commonly known, the anticancer mechanism of Se is related to its significant antioxidant capacity [20]. Razaghi et al. noticed that Se status in cancer patients is highly correlated with concentrations of proinflammatory cytokines [18]. Varlamova and Turovsky described the main cytotoxic activities of methylseleninic acid on different cancer cells [81]. 

Considering that cancer cells are quite vulnerable to exposure to ROS, targeting the antioxidant capacity of tumor cells has been considered a promising strategy for anticancer therapy [82]. At increased but non-lethal doses, Se acts as a pro-oxidant and inhibits the growth of cancer cells without side effects on normal cells [53,83]. According to Wang et al., the detection by flow cytometry has shown decreased cancer cell viability of buffalo rat liver induced by Se nanoparticles (over 24 μM) [84]. It occurred mainly due to tumor cell apoptosis rather than necrosis. Thus, Se was regarded as a ‘double-edged sword’ due to its antioxidant properties at nutritional levels or prooxidant effects at supranutritional levels [18,85]. In tumor cells, due to the acidic pH state with redox imbalance, excessive Se-compounds cause ROS overproduction, leading to ER stress and disruption of the integrity of mitochondria [79]. The anti-cancer effects of Se-compounds are related to their ability to induce oxidative stress and subsequent DNA damage in cancer cells that lead to obligate apoptosis [17]. On the contrary, the Se intake at an optimal level could prevent damage to the DNA in healthy cells and, consequently, the occurrence of mutations.

As it is known, benign prostatic hyperplasia and adenocarcinoma are closely associated with the age of men. Daragó et al. found that deficiency of Zn, Cu, and Se caused disturbing homeostasis in the etiology of the abovementioned prostatic diseases [86]. Se nanoparticles with a size below 100 nm produced by a novel green process called pulsed laser ablation in liquids (PLAL) showed an anticancer effect on human glioblastoma and melanoma cells [62]. Se-nanoformulations’ anticancer effects inhibited cancer cells’ growth by interrupting the cell cycle at the synthetic phase [87]. Selenomethylselenocysteine, found in Se-enriched plants from the *Brassica* and *Allium* genera, was regarded as a compound possessing prominent anti-cancerous properties [25].

During the clinical study, 325 cases of Chinese patients with oral cancer were analyzed by Chen et al. [88]. The multiple interactions between Se intake, drinking/smoking status, and fish and fresh fruit intake frequencies were studied. The high level of serum Se was regarded as a protective factor for the risk of oral cancer. Appropriate diet and immunity are considered important modifiable factors in oral cancer [89]. Se and ceruloplasmin levels in serum were regarded as disease markers in squamous cell carcinoma. The effect of Se on the reduction of all-cause and cancer mortality was observed in the 12-year study carried out on 13,887 adults in the US population [90].

### 2.5. Intoxication

Se is an effective protective agent for the human body against various environmental pollutants and drug-related side effects [91]. Limaye A. et al. found that Se and polyphenol curcumin were quite effective against aflatoxicosis due to their prominent antioxidant properties [92]. Bjørklund [93] found that numerous studies have shown that many Se-containing foods protect the human body against mercury (Hg) exposure. It is due to the high affinity between Se and Hg. The appropriate intake of Se and Zn and some vitamins have been suggested to reduce As-induced toxicity [94].

## 3. The Role of Se in Longevity and Age-Related Disorders

Age-related disorders such as neurodegeneration, cardiovascular disease, immune dysfunction, formation of skin wrinkles, etc., are closely associated with Se deficiency [95]. Se concentration in healthy adults’ plasma is higher than in the elderly population [96].

The items related to the possible effects of Se application for increasing animals’ longevity were widely investigated [97,98,99,100]. The activity of GSH-Px in the female and male flies *Drosophila melanogaster* was elevated with the increased sodium selenite in the medium. The average life span and the average maximum life span of flies in the Se groups improved significantly compared with the control group [100]. Another animal study showed that the survival rate of flies fed a diet deficient in Se was one-half that of flies fed a diet supplemented with optimal amounts of Se [99]. Administration of sodium selenite for seven days significantly increased the longevity of mice inoculated with 10(5) L797 cells. These outcomes suggest that the anti-leukemic effect of sodium selenite is associated with the inhibition of DNA replication, transcription, and translation [97]. In their recent publication, Wu et al. suggested a novel aging model by which Se at low levels may be considered a hormetic chemical and decouple health span and longevity [22]. The authors’ experimental data revealed that dietary Se deprivation increased the incidence of osteoporosis, gray hair, alopecia, and cataract but surprisingly promoted longevity in mice; deprivation also accelerated age-dependent declines in glucose tolerance, insulin sensitivity, and glucose-stimulated insulin production.

Steinbrenner and Klotz analyzed the epidemiological studies [101]. They revealed that the insufficient dietary intake of Zn and Se can cause cognitive dysfunctions in aged persons. Se supplementation effectively restored cognitive decline associated with aging [2]. Even though Se is crucial to brain health, at the same time, it can exhibit neurotoxicity, depending on speciation and dosage [102]. The valuable skincare and anti-aging effects were recorded by Wei et al. after the external application of Se-enriched fermented mung beans [103].

Ischemic heart death correlated inversely with blood Se in 25 US cities in 22 states [104]. The association between serum Se and hand grip strength among 676 moderately to severely disabled community-dwelling women in Baltimore, Maryland, was established [105]. Population-based studies showed that low serum Se and total carotenoid concentrations were associated with an increased risk of death among older women in the US population [106]. According to Al-Mubarak et al., insufficient Se intake was discovered in 30–50% of patients with heart failure [107].

Body Se in centenarians is maintained at a nutritionally adequate level, suggesting a positive association between selenoprotein expression and longevity. Elevated ROS levels contributed to the pathologies of Alzheimer’s and Parkinson’s diseases, which antioxidative selenoproteins can suppress. Selenoprotein p (SelP) is essential for the normal function of neuronal cells and protects against Alzheimer’s disease [22].

Scientists conducted several investigations concerning environmental and nutritional factors, including Se consumption, relating to human longevity in Chinese areas [104,108,109,110,111,112]. In 446 oldest elderly from longevity areas in China (5 provinces), the median (interquartile range) of the content of plasma Se was 1.44 (0.91) μmol/L. The contents of plasma Se, Fe, and Cu in centenarians were higher than in those aged 90 and over; the contents of plasma Se increased with age. The concentrations of plasma Se were high in the oldest elderly in the longevity areas [111]. The centenarians from seven longevity areas in China, who participated in another longitudinal survey [111], had lower chronic disease risks and higher antioxidant activity compared with other age groups and had a higher level of nutritional elements compared with those aged 90 and over. A cross-sectional study [108] in all 18 cities and counties in Hainan Province revealed a positive correlation between daily intake of Cu, Se, and Zn from food and water and aging and longevity indexes. Another Chinese study confirmed that the percentage of long-lived people in the Zhongxiang area, where the inhabitants commonly have long life spans, was closely related to the macro- and microelement contents of their staple food, rice [110]. The study’s authors classified the elements of rice based on their effect on longevity, indicating that Se showed a positive correlation with it. Foster and Zhang determined that fewer people of advanced age reside in Chinese counties where Kaschin-Beck and Keshan diseases are endemic than in unaffected counties [112]. These researchers substantiate it by elevated mortality from endemic and chronic diseases in Se-deficient areas and accelerated aging due to excessive cellular damage. In Se deficient areas of China, the life span of adults was lowered severely, with the occurrence of heart muscle damage [104]. Huang et al. [113] found higher 85+/65+ distribution ratios, indicating enhanced longevity in the coastal southern and eastern regions of China, whereas higher levels of Se distribution in soil occur [109]. Nutritional factors such as Se and omega-3 fatty acids in sea fish were crucial to longevity there.

A cohort study of 227 older adults residing in 14 nursing homes in Asturias (Spain) revealed that subjects with the upper tertile of serum Se had more than twice as much probability of reporting good health status, good chewing ability, and of doing more than 60 min of exercise/day [38]. Researchers who designed a 9-year longitudinal EVA study in France involving 1389 free-living participants aged 59–71 years [37] investigated the relationships between plasma Se and longevity. They concluded that inadequate plasma Se could adversely affect the maintenance of optimal health in an aging population. Comparisons of survival distributions among quartiles of plasma Se showed that mortality increased in subgroups with low plasma Se concentrations at baseline; a 0.2 µmol/L decrease in plasma Se was significantly associated with higher mortality risk.

The low percentages of Se deficiencies were reported as a possible explanation for longevity in Italian scientists’ nonagenarian–centenarian studies. A significant decrease in Se values was demonstrated in a group of nonagenarians/centenarians (91–110 years) compared to a group of elderly subjects (60–90 years). Another study [114] involved subjects who lived in Sardinia, an Italian island with a higher prevalence of centenarians than in other European countries. Significant depletion of Se concentration in plasma of nonagenarians (89.0 ± 6.3 years) and centenarians (101 ± 1 years) with respect to controls (61.2 ± 1.1 years) was shown; the geometric mean values of Se were: 111 µg/mL in controls, 88.9 µg/mL in nonagenarians, 81.9 µg/mL in centenarians.

## 4. Sources of Se in the Human Diet

**Figure 2:**
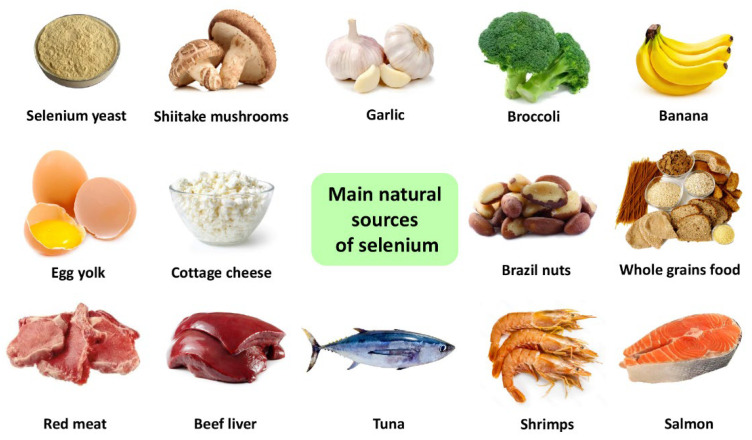
The key sources of Selenium from food.

Organic Se from food is considered a safe and efficient source of supporting human health [115]. Among the sources of organic Se for humans, we find foods of animal, vegetable, and mushroom origin [13,116]. The main animal sources of Se are red meats, poultry, beef or sheep liver, seafood, eggs, and dairy products [31,35,117]. The main kinds of food containing Se in comparatively high amounts are shown in Figure 2**.**

Plants can absorb inorganic Se from soil and transform it into an organic form such as selenomethionine or selenocysteine, which are much more accessible for animals and humans than inorganic ones [25,118]. Consumed by humans, organic Se changes by joining amino acids and proteins [25].

The extremely high Se content, mainly in the form of selenomethionine, is a characteristic feature of Brazilian nuts [20]. Broccoli, which can accumulate Se many-fold higher than other plants, is associated with a reduced risk of some cancer types [119]. Consumption of Se-enriched broccoli led to activation of human leukocytes and increased cytokine production during immune response [120]. It should be noted that consumed vegetables (garlic, broccoli, etc.) should contain methylated forms of organic Se to be effective in the prevention of cancer [25,117]. Methylseleninic acid can cause stress in the functioning endoplasmic reticulum through modulation of the membrane selenoproteins and activating the apoptosis of cancer [52,61,121].

The cluster analysis of average Se amounts in raw material of different medicinal plants demonstrated that the *Apiaceae* and *Lamiaceae* representatives are more Se-rich than species from other botanical families [122]. Thus, Majoranae herba (*Lamiaceae*) contained more than 50 μg/kg Se. An organic Se-compounds from mushroom *Grifola frondosa* formed by its combination of polysaccharides was very effective in immune regulation and had antitumor and anti-aging effects [123]. Se-containing yeasts are a valuable source of easily assimilable Se [20]. Fordyce reported that the bioavailability of Se was reduced in dairy products, mushrooms, and vegetables as a result of cooking, which causes losing about 50% of Se compounds, especially when vinegar and salt are added [124].

In recent decades, biofortification strategies have been widely applied to produce Se-enriched edible plants [125]. Enrichment of soil with fertilizers such as inorganic Se compounds leads to a rapid increase in crops’ organic Se levels [118]. It was also demonstrated that the plant biomass, bacteria, and yeast enriched in Se from the culture medium containing its inorganic sources were considered a prospect for this trace element supplementation [126]. Se-enriched yeast is the cheapest organic Se supplementation [25]. The concentration of Se in plants usually reflects its soil content and can vary in a very wide range, from 0.005 mg/kg to 5500 mg/kg [13,127]. Se-containing yeast used as a feed supplementation for livestock is currently approved in European countries to enrich animal food with this element [128].

## 5. Nanoformulations of Se in Age-Related Disorders

Nanoscale Se provides increased bioavailability and controlled release of various drugs into the body at the site of action. [129,130]. Nanoparticles supply a low dosage to restore the Se serum levels [131]. Various studies demonstrated that Se nanoparticles possess lower toxicity and higher bioavailability than organic or inorganic Se [84,132]. They are characterized by high adsorption capacity as the charges on their surface can conjugate and interact through various functional groups (C=O, COO−, NH, C–N, etc.), both positively and negatively charged [133].

Se nanoparticles with chitosan/citrate complex have protected experimental mice against ROS in galactose-induced aging [134]. Mamgain et al. reported the antioxidant activity of diverse Se-based nanoparticles [135]. The antioxidant, anti-carcinogenic, and immunomodulatory efficacy of nano-Se applications for enhancing stress resilience and productivity of livestock and fish were proven by Sarkar et al. [136]. The activation of oxidative stress in cancer cells was regarded as a key point in treating oncological disorders with Se nanoparticles [79].

Varlamova et al. summarized the data of many researchers who described the positive physiological effect of nano-scale Se in the case of neurodegenerative disorders [79]. Nano-Se dietary supplementation improved the Se deposition in the testis and ovary of Japanese quail and their reproductive performance, thus prolonging their youth [137]. Se nanoparticles effectively prevented the progression of diabetic nephropathy [138]. Se nanosupplementations reduced the formation of advanced glycation end products by slowing down the process of protein glycation [139]. Se-nanoparticles demonstrated valuable cytotoxic potential in treating cancer in different cell lines [140,141]. Wang et al. reported that nanoscale Se supplementation possesses significant chemopreventive properties [142]. Turovsky and Varlamova comprehensively described the mechanism of calcium-dependent pro-apoptotic action of Se nanoparticles [143]. It was concluded that instead of systemic supplementation of Se, nano-formulations could be developed for targeted delivery of Se to tumors and decrease its dosage to minimize the adverse side effects [18,144].

## 6. Determination of Se in Foods

Se appears in the environment in a trace amount. Thus, determining the Se amount in food samples demands appropriate measurement methods, including sample preparation, separation technique, and detection [25]. Se speciation in food is not an easy goal because of its very low concentrations and availability of many different forms, as well as the lack of appropriate reference materials for its speciation that generates problems with validation. That is why speciation of Se has been conducted only for a few foods [25,145].

Bodnar et al. summarized that among different sample preparation techniques for Se determination, cryogenic trapping, wet/dry mineralization, and extraction methods (solid-phase, liquid–liquid, liquid–solid, and enzymatic) are the most used [25]. A UV-oxidation procedure was developed to completely digest food samples for the evaluation of trace levels of Se [146].

The chromatographic methods (liquid and gas chromatography) are more frequently used than capillary electrophoresis or isotachophoresis in the field of separation techniques. Gilbert-López et al. reported that more than 100 Se metabolites were identified in the Se-enriched yeast using the liquid chromatography-mass spectrometry method [147]. Among the detection systems, spectroscopic, spectrophotometric, and electroanalytical methods are mostly applied [25,148,149]. Thus, the detection limits for Se in the plant food using atomic absorption spectrometry were 35–40 ng/g [146].

## 7. Conclusions

The oxidative damage to macromolecules in the human body represents a suitable environment for developing age-related diseases. The trace element Se in the form of selenoproteins improves antioxidant defense, immune functions, and metabolic homeostasis. Se deficiency affects about one billion people in the world and may have a significant adverse effect on human health. Se-containing organic compounds play a key role in the health maintenance of aging individuals. The nutritional doses of Se can efficiently stimulate the immune system against infectious diseases or cancer. A deficiency in Se is evidenced in some disorders caused by viruses or pathogenic bacteria. It discusses the important roles of Se-enzymes according to their involvement in the thyroid hormones’ metabolism. Insufficient dietary intake of Se can cause cognitive dysfunctions and heart failure in aged persons. Organic Se from food of animal, plant, or mushroom origin is considered a safe and efficient source of supporting human health. Bio-fortification strategies are widely applied to produce Se-enriched edible plants and mushrooms. Nanoscale Se provides increased bioavailability and controlled release in the organism to the site of action. Generally, the health effect of Se is dose-dependent, and there is a narrow margin between its essential levels and the amounts associated with toxicity. Se possesses antioxidant properties at nutritional levels and prooxidant effects at supranutritional levels.

## Data Availability

Not applicable.
